# Modulation of extracellular matrix genes reflects the magnitude of physiological adaptation to aerobic exercise training in humans

**DOI:** 10.1186/1741-7007-3-19

**Published:** 2005-09-02

**Authors:** James A Timmons, Eva Jansson, Helene Fischer, Thomas Gustafsson, Paul L Greenhaff, John Ridden, Jonathan Rachman, Carl Johan Sundberg

**Affiliations:** 1 Department of Physiology and Pharmacology, Karolinska Institutet, Stockholm, SE171 77, Sweden; 2 Centre for Genomics & Bioinformatics, Karolinska Institutet, Stockholm, SE171 77, Sweden; 3 Laboratory Medicine, Division of Clinical Physiology, Karolinska Institutet, Huddinge, 141 86, Sweden; 4 Centre for Integrated Systems Biology and Medicine, University Medical School, Nottingham, UK; 5 Department of Enabling Technologies, AstraZeneca, Alderly Park, UK; 6 OSI Prosidion Ltd, Oxfordshire, OX4 6LT, UK

## Abstract

**Background:**

Regular exercise reduces cardiovascular and metabolic disease partly through improved aerobic fitness. The determinants of exercise-induced gains in aerobic fitness in humans are not known. We have demonstrated that over 500 genes are activated in response to endurance-exercise training, including modulation of muscle extracellular matrix (ECM) genes. Real-time quantitative PCR, which is essential for the characterization of lower abundance genes, was used to examine 15 ECM genes potentially relevant for endurance-exercise adaptation. Twenty-four sedentary male subjects undertook six weeks of high-intensity aerobic cycle training with muscle biopsies being obtained both before and 24 h after training. Subjects were ranked based on improvement in aerobic fitness, and two cohorts were formed (n = 8 per group): the high-responder group (HRG; peak rate of oxygen consumption increased by +0.71 ± 0.1 L min^-1^; p < 0.0001) while the low-responder group (LRG; peak rate of oxygen consumption did not change, +0.17 ± 0.1 L min^-1^, ns). ECM genes profiled included the *angiopoietin 1 *and related genes (*angiopoietin 2*, *tyrosine kinase with immunoglobulin-like and EGF-like domains 1 (TIE1) and 2 *(*TIE2*), *vascular endothelial growth factor *(*VEGF*) and related receptors (*VEGF receptor 1, VEGF receptor 2 and neuropilin-1*), *thrombospondin-4*, *α2-macroglobulin *and *transforming growth factor β2*.

**Results:**

*neuropilin-1 *(800%; p < 0.001) and *VEGF receptor 2 *(300%; p < 0.01) transcript abundance increased only in the HRG, whereas levels of *VEGF receptor 1 *mRNA actually declined in the LRG (p < 0.05). *TIE1 *and *TIE2 *mRNA levels were unaltered in the LRG, whereas transcription levels of both genes were increased by 2.5-fold in the HRG (p < 0.01). Levels of *thrombospondin-4 *(900%; p < 0.001) and *α2-macroglobulin *(300%, p < 0.05) mRNA increased substantially in the HRG. In contrast, the amount of *transforming growth factor β2 *transcript increased only in the HRG (330%; p < 0.01), whereas it remained unchanged in the LRG (-80%).

**Conclusion:**

We demonstrate for the first time that aerobic training activates *angiopoietin 1 *and *TIE2 *genes in human muscle, but only when aerobic capacity adapts to exercise-training. The fourfold-greater increase in aerobic fitness and markedly differing gene expression profile in the HRG indicates that these ECM genes may be critical for physiological adaptation to exercise in humans. In addition, we show that, without careful demonstration of physiological adaptation, conclusions derived from gene expression profiling of human skeletal muscle following exercise may be of limited value. We propose that future studies should (a) investigate the mechanisms that underlie the apparent link between physiological adaptation and gene expression and (b) use the genes profiled in this paper as candidates for population genetic studies.

## Background

Regular exercise and high aerobic fitness both reduce the risk of cardiovascular- and metabolic-disease-related death for a multitude of potential reasons [1-5]. It is noteworthy that a very large intersubject variation exists when measuring the physiological adaptation to supervised exercise training [6-9]. While some subjects demonstrate a robust increase in aerobic capacity, others seem not to respond substantially at all [8,10,11]. This variation also applies to the improvement in insulin sensitivity seen after exercise [9]. Such observations may be important for future cardiovascular health, as an inherent lack of 'trainability' associates with increased cardiovascular risk factors [5]. The mRNA abundance for a huge number of genes (>500) have been shown to be increased many hours after exercise training in humans [8,12-18]. However, only very recently have gene expression changes been related to the magnitude of physiological adaptation [8,9]. Teran-Garica and others [9] observed a divergent mRNA response in subjects that increase their insulin sensitivity most following endurance training, whereas we have demonstrated that the expression of *insulin-like growth factor *related genes were increased with training and more markedly in those subjects that most enhanced their aerobic capacity [8]. Little else is known about the relationship between the extent of gene activation and the magnitude of physiological adaptation to exercise training in humans.

Increased aerobic capacity following a period of intense endurance training reflects both central and peripheral adaptations [19-23]. Activation of angiogenesis is presumably an important component of the response to endurance training [15,20,23-25], indicating that substantial remodeling of skeletal muscle extracellular matrix (ECM) is required. Using gene expression profiling, our knowledge of the many factors that regulate the extracellular environment and facilitate vascular remodeling following exercise has improved. Alterations in *vascular endothelial growth factor *(*VEGF*) and related receptor(s) transcript expression occur following acute exercise and endurance training [15,17,26-28], indicating that VEGF may be an important exercise factor. More recently, the Angiopoietin (ANG) signaling pathway has been shown to synergize with VEGF [29,30] and expression of both *ANG1 *and *ANG2 *is altered by intense aerobic training in rats [27]. There is currently no information on the physiological regulation of the ANG system in human skeletal muscle following aerobic training. Furthermore, the significance of changes in the abundance of transcripts from genes for various growth factors [15,26,27] has not been examined in the context of changes in aerobic capacity resulting from endurance training.

We therefore set out to establish if greater improvements in systemic cardiovascular adaptation would be associated with changes in muscle gene expression. We did so by examining the expression responses of a number of tissue remodeling genes in subjects that demonstrated either a substantial (high-responder group; HRG) or a modest/negligible (low-responder group; LRG) response to training [8]. We also aimed to establish if the inclusion of low responders in a gene analysis study could yield misleading information on genes that might genuinely contribute to physiological adaptation to exercise in humans.

## Results

### Physiological parameters

Table 1 presents the baseline physiological characteristics of the HRG and LRG. As can be observed, no differences in baseline demographics or physiological characteristics existed between LRG and HRG subjects prior to the training program [8]. The combined training response was significantly greater in the HRG (p < 0.001). The reduction in submaximal heart rate was more substantial (p < 0.01) in the HRG (-25.9 ± 3.6 beats min^-1 ^(BPM)) vs. the LRG (-10.5 ± 3.5 BPM) during a 10 min fixed-workload, submaximal cycle. The increase in work performed during 15 min cycling after the six weeks of endurance exercise training was 60% greater (p < 0.05) in the HRG (+40.8 ± 4.3 kJ) than in the LRG (+24.9 ± 4.1 kJ). The increase in peak rate of oxygen consumption (peak VO_2_) was four times greater (p < 0.001) in the HRG (+0.71 ± 0.1 L min^-1^) than in the LRG (+0.17 ± 0.1 L min^-1^). Importantly, there was no correlation between baseline physiological characteristics and the magnitude of improvement noted; this observation is consistent with those from previous studies [31].

### Growth factor-related genes

**Figure 1:**
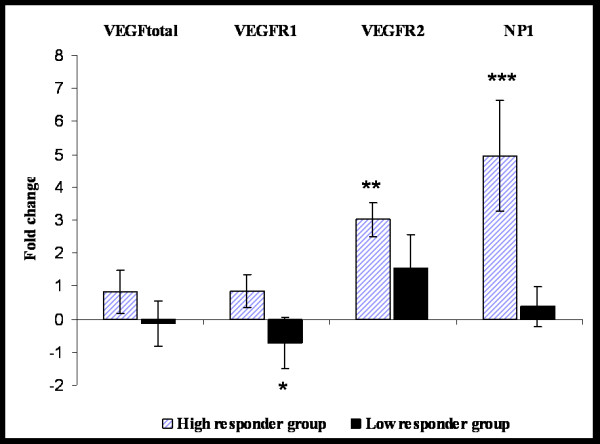
**Change in Vascular endothelial growth factor-related gene expression following endurance training. **Values are -fold changes in human skeletal muscle gene expression (mean ± SE) following six weeks of aerobic training. Gene expression was determined using real-time quantitative PCR. Following six weeks training (n = 24), the eight highest and eight lowest responders to exercise training were identified using the sum of (a) the percent improvement in maximal aerobic capacity, (b) the percent reduction in submaximal heart rate during 15 min fixed-workload, submaximal cycling and (c) the percent improvement in work done during a 15 min maximal cycling test. This ranking was carried out before any genomic analysis was carried out. The training responses were evaluated by two-way ANOVA and Bonferoni post-hoc tests. * indicates p < 0.05, ** indicates p < 0.01 and *** indicates p < 0.001.

**Figure 2:**
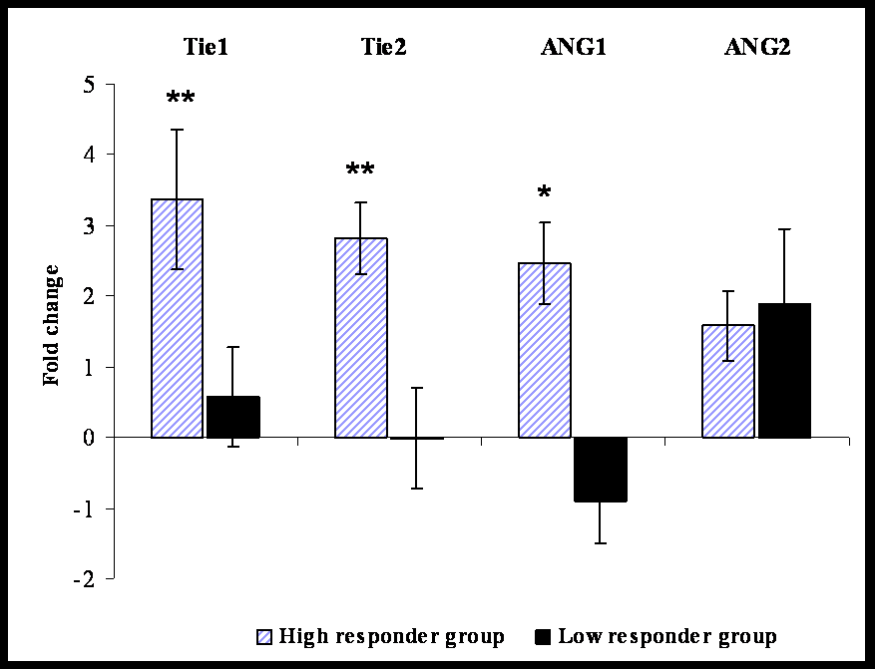
**Changes in Angiopoietin-related gene expression following endurance training. **Values are -fold changes in human skeletal muscle gene expression (mean ± SE) following six weeks of aerobic training, as described in Fig. 1. * indicates p < 0.05, ** indicates p < 0.01 and *** indicates p < 0.001.

**Figure 3:**
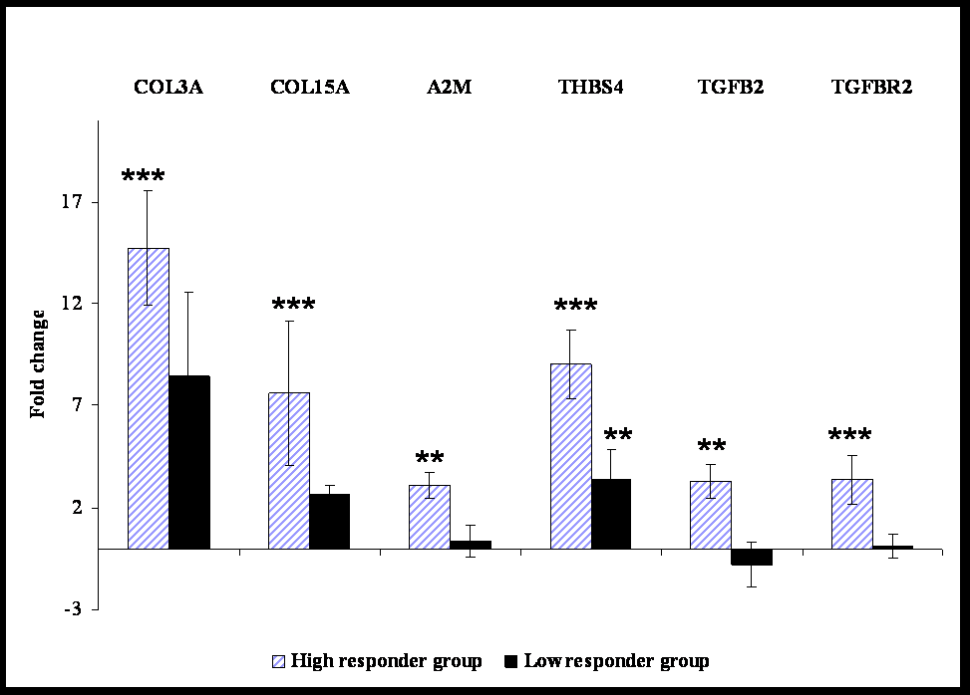
**Change in extracellular-matrix-related gene expression following endurance training. **Values are -fold changes in human skeletal muscle gene expression (mean ± SE) following six weeks of aerobic training, as described in Fig. 1. * indicates p < 0.05, ** indicates p < 0.01 and *** indicates p < 0.001.

Subjects in both groups underwent six weeks of endurance training. Gene expression levels were then measured 24 h after the last training session. Levels of *VEGF *gene expression were not significantly altered in either group (Fig. 1). Likewise, levels of *VEGF receptor 1 (VEGFR1) *mRNA did not significantly increase in the HRG and actually declined in the LRG (p < 0.05). However, *VEGF receptor 2 *(*VEGFR2) *mRNA expression increased by threefold in the HRG (p < 0.01), whereas it did not significantly change in the LRG. Similarly, mRNA for the VEGFR2 coreceptor Neuropilin-1 (NP-1) increased in the HRG (p < 0.001) but not in the LRG (Fig. 1). Expression of the *hypoxia-inducible factor 1α *(*HIF*) gene did not significantly change after endurance training. Regulation by endurance training of *ANG*-related genes was only observed in the HRG (Fig. 2). Levels of mRNA coding for ANG1, an agonist for the Tyrosine kinase with immunoglobulin-like and EGF-like domains 2 (TIE2) receptor, increased significantly in the HRG (p < 0.05), although *ANG2 *levels did not change significantly in either group. Likewise, transcription of *tyrosine kinase with immunoglobulin-like and EGF-like domains 1 *(*TIE1*; 3.4 ± 1.0-fold increase; p < 0.01) and *TIE2 *increased in the HRG only (p < 0.01; Fig. 3).

### Extracellular matrix growth factor binding genes

Using our microarray dataset [8], we selected ECM factors that (a) demonstrated evidence of being modulated by exercise training and (b) were relevant for ECM remodeling [32-34]. These genes included ones that encoded structural components of blood vessels (*collagen type IIIα1*) or known regulators of tissue angiogenesis (*collagen type XVα1*), factors known to influence ECM-derived growth factor activity (*α2-macroglobulin (A2M) *and *thrombospondin-4 (THBS4)*), *transforming growth factor β2 *(*TGFB2*; a potent regulator of tissue remodeling) and *TGFB receptor II *(*TGFBR2*). The level of fetal vascular collagen (i.e., *collagen type IIIα1*) gene expression was increased 14.7 ± 2.8-fold (p < 0.001) in the HRG and 8.5 ± 4.1 fold in the LRG (p < 0.001). The level of *collagen type XVα1 *expression increased in both groups: the HRG demonstrated a 7.6 ± 3.6-fold increase (p < 0.001) while the LRG demonstrated a 2.7 ± 0.5-fold increase (p < 0.01) in expression of the gene (Fig. 3). In contrast, while both *A2M *and *THBS4 *were significantly upregulated in the HRG (threefold (p < 0.01) and tenfold (p < 0.001) increases, respectively), in the LRG the *THBS4 *response was more modest (2.5-fold; p < 0.01) and *A2M *mRNA levels did not significantly change. *TGFB2 *was significantly upregulated in the HRG (p < 0.01), whereas, if anything, it tended to be downregulated in the LRG (Fig. 3). The level of *TGFBR2 *expression was unchanged in the LRG, but threefold increased in the HRG (p < 0.001; Fig. 3). Finally, three genes unrelated to ECM biology but previously described as being modulated by exercise [8], *interleukin 17D*, *Rho-GTPase-activating-protein 1 *and *myristoylated alanine-rich protein kinase C substrate*, did not significantly vary in their expression between HRG and LRG (data not shown).

## Discussion

Classic alterations in skeletal muscle phenotype following physical training include improved fatigue resistance, enhanced aerobic capacity and greater insulin sensitivity [9,23,25,35,36]. The significance of an individual's ability to adapt to exercise training may ultimately influence multiple risk factors important for long term cardiovascular health [5]. In the present study we demonstrate for the first time that *ANG *genes are substantially modulated in humans following a six-week aerobic training program. Overall, many ECM-gene transcripts were only modulated in subjects that demonstrated a concurrent improvement in aerobic capacity. Our data suggest that activation of ECM genes may help determine the cardiovascular adaptation to aerobic exercise in humans. The present findings also indicate that, when carrying out expression studies of gene transcripts in humans, prior to any interpretation of muscle gene expression responses, attention must be afforded to the presence of physiological adaptation.

### Modulation of genes that regulate extracellular matrix remodeling

We still have an incomplete understanding of the endogenous processes that regulate physiological adaptation to aerobic exercise. Vascular growth factors not only regulate tissue blood vessel density, but also enhances the expression of proteins that regulate oxygen levels in tissue [37]. Hence, it is naïve to think about the role of such growth factors only in terms of regulating tissue capillary density. Altered expression of HIF responsive genes (e.g. *VEGF *or *VEGFR1*) typically reflects the posttranslational stabilization of HIF1α protein [38], and consistent with this dogma, *HIF *mRNA was not significantly upregulated. *VEGFR2*, considered the major mediator of VEGF-A-related angiogenesis, was significantly upregulated in the HRG only. Furthermore, *NP-1*, a facilitator of VEGF_165 _action at VEGFR2, was markedly elevated in the HRG and unchanged in the LRG (Fig. 1). Upregulation of the VEGFR2 coreceptor transcript provides some evidence for greater VEGF activity in the HRG, as enhanced *NP-1 *gene expression can be mediated by VEGF signaling [39,40]. The significance of the downregulation of *VEGFR1 *in the LRG is unclear, but plausibly reflects an (unsuccessful) compensation for the general lack of *VEGFR2/NP-1 *gene activation in the LRG, as studies indicate VEGFR1 may oppose VEGF signaling via VEGFR2 in some situations [41].

The present study represents the first characterization of expression levels of the human *ANG *gene family in response to endurance-exercise training. Recently, the Terjung laboratory examined the impact of exercise on the angiopoietin system in rodents [27]. Alterations in *TIE2*, *ANG1 *and *ANG2 *transcript expression were profiled 2 h post exercise in various muscle groups taken from Sprague-Dawley rats after 1 to 24 days of intense aerobic running [27]. In rodents, activation of *TIE2 *and *ANG2 *was, broadly speaking, rather similar across each muscle tissue, especially when one considers that recruitment patterns would differ between the various muscle groups studied. The *ANG1 *response, however, differed both across muscle groups and when compared with our human data. In contrast with the upregulation of *ANG1 *seen in humans in the HRG (Fig. 2), in the rat *ANG1 *expression was slightly downregulated in oxidative muscle groups, while 'fast' gastrocnemius demonstrated only a modest increase in *ANG1 *expression [27]. It has recently been established that either pre- [30] or concurrent administration [29] of ANG1 synergizes with VEGF to promote hindlimb angiogenesis. Thus it makes sense that effective aerobic training might result in stable increases in *ANG1 *expression (as we have found). Therefore, differences between our human data and the rodent study by Lloyd et al [27] perhaps reflect differing muscle sampling times. Future human studies should ideally use multiple time points post exercise to clarify these issues. However, care should be taken to verify that the subjects studied are able to demonstrate a measurable aerobic training response otherwise such results may be unreliable.

It has been hypothesized that changes in the ratio between levels of ANG1 and ANG2 is of physiological importance [27,42] by contributing to the stabilization of the primary endothelial structures [43]. While ANG1's ability to antagonize ANG2 at the TIE2 receptor may be cell-type specific [42,44,45] and has yet to be proven to be important *in vivo*, it is interesting to note that ANG1 and ANG2 appear to have identical binding affinities for the TIE2 receptor, whereas a threefold molar excess of ANG2 is required to antagonize ANG1 activity [45]. If the gene expression patterns observed in the present study translate to greater Tie2 receptor signaling, then our data supports the idea that ANG1 cooperates with other growth factors *in vivo *to regulate and promote functional angiogenesis. This hypothesis clearly requires further investigation. ANG1 and TGFB signaling facilitate the maturation of VEGF-stimulated collateral vessel growth in adult tissue [29,30,46]. Notably, TGFB2 and TGFBR2 were substantially upregulated only in the HRG. This observation again suggests that the ECM-related gene response in the HRG may have allowed for greater tissue remodeling, which would have contributed to the fourfold greater increase in aerobic capacity.

To further examine the idea that the pattern of transcript expression in the HRG contributed to the enhanced aerobic adaptation, we profiled a second set of genes chosen from a gene-array study [8]. For example, upregulation of potent growth factors should be accompanied by upregulation of endogenous regulators, so that physiological control could be maintained. A2M is an ECM protein known to bind and regulate growth factor activity [47,48]. *A2M *was substantially upregulated in the HRG only (Fig. 3) suggesting that there was more active growth factor signaling within the ECM of the HRG. Thrombospondins also regulate ECM growth factors, including TGFB activity. In addition, a loss of function polymorphism in *THBS4 *is strongly associated with premature coronary artery disease [49]. In the present study a more substantial increase in *THBS4 *mRNA expression was noted in the HRG (Fig. 3). As dysfunction of *THBS4 *and lack of cardiorespiratory fitness are both risk factors for cardiovascular disease, it is plausible that *THBS4 *plays a role in the cardioprotective effects of exercise. Further analysis is required to establish if such a relationship exists within a larger human population.

## Conclusion

There was unquestionably a differential physiological response between the groups, as the HRG increased their aerobic capacity by, on average, 0.71 L min^-1^, whereas the LRG did not significantly increase their aerobic capacity (+0.17 L min^-1^). At this time, our data cannot directly attribute cause-and-effect for the differentially responding genes. Although we profiled changes in skeletal muscle ECM-related gene expression changes, we are not implying that only the local (i.e., in skeletal muscle) role of these genes contributes to the magnitude of the training adaptation. It is entirely plausible that the observed difference between our cohorts reflects a genotype-dependant response, which would impact on gene expression in a range of tissues important for cardiovascular adaptation to endurance-exercise training. Importantly, histological analysis of muscle would not address this possibility. Instead, we would suggest that these differentially expressed genes represent reasonable candidates for future polymorphism studies in larger populations. Our data also demonstrates that direct evidence for physiological adaptability must be presented prior to concluding that gene transcript alterations may or may not occur during the physiological adaptation to exercise.

## Methods

### Human and physiological measurements

The study was approved by the ethics committee of the Karolinska Institutet, Stockholm, Sweden, and informed consent was obtained from each of the volunteers. Subjects abstained from strenuous exercise during the three weeks prior to obtaining pretraining muscle biopsies (taken from the vastus lateralis). Twenty-four subjects trained under full supervision on a cycle ergometer four times a week (45 min) at 75% of their pretraining peak VO_2 _for six weeks. Posttraining biopsies were taken 24 h after the last training session. Physiological measurements and muscle biopsies were performed as previously described [8,16,50]. All physiological parameters were derived from a minimum of two assessments, taken on separate days. Peak VO_2 _was determined using a cycle ergometer (Rodby, Sweden). An incremental protocol was combined with continuous analysis of respiratory gases using the Sensormedic ventilator (Sensormedic Co., USA). At peak VO_2 _the respiratory exchange ratio and heart rate exceeded 1.1 and 190 BPM, respectively, on all occasions. Total amount of work done in 15 min of cycling was determined using a self-paced protocol (Lode, Netherlands, test-re-test variability <5%). Submaximal physiological parameters were determined during two separate 10 min constant-load, submaximal cycling sessions (carried out at 75% of pretraining peak VO_2_).

Two groups were selected from a larger training cohort based on the magnitude of their training response (Table 1). The two groups represented the eight subjects that demonstrated the largest improvement in physiological capacity (HRG) and the eight subjects that demonstrated minimal changes in physiological capacity (LRG) after following an identical supervised training program. The average training-induced change in peak VO_2 _in the LRG equates to the lower ~10% of responses observed in the HERITAGE study [6], whereas the response in the HRG represents the top ~10% of responses. This information helps to establish a 'population perspective' on the responses observed in the present study. Subjects were assigned to each group after being ranked on the basis of the sum of changes in three main physiological parameters: (a) percent improvement in peak aerobic capacity, (b) percent reduction in submaximal heart rate during 10 min fixed-workload, submaximal cycling and (c) percent improvement in work done during a 15 min maximal cycling test. A combination of physiological markers of adaptation was used to ensure that no spurious individual physiological measurement would compromise the overall categorization of a subject [8]. Peak aerobic capacity, decreased exercise-induced-increases in heart rate and aerobic performance are all valid and accepted responses to aerobic exercise training. No molecular or biochemical analysis was carried out until the subjects were assigned to their particular group. This type of novel analysis strategy has previously been used by our group and also, more recently, by Bouchard et al [8,9]

### Real-time quantitative PCR and statistics

Total RNA was prepared using the TRIzol method (Invitrogen, USA) and quantified using a spectrophotometer. Two μg of RNA was reverse transcribed by Superscript reverse transcriptase (Life Technologies, Sweden) using random hexamer primers (Roche Diagnostics GmbH, Mannheim, Germany) in a total volume of 20 μl. Detection of mRNA was performed using a ABI-PRISM^® ^7700 Sequence Detector (Perkin-Elmer Applied Biosystems Inc, Foster City, CA, USA). All reactions were performed in 96-well MicroAmp Optical plates (Perkin-Elmer Applied Biosystems Inc.). Amplification aliquots contained 5 μl of the sample cDNA, the TaqMan Universal PCR master mix (Perkin-Elmer Applied Biosystems Inc.) and an optimized concentration of each primer and probe, prepared according to the manufacturer's recommendation, in a final volume of 25 μl. 18S rRNA was selected as an endogenous control to correct for potential variations in RNA loading into the cDNA synthesis reaction. The 18S rRNA control was run in triplicate in separate wells (using 1:2000 dilution of the original cDNA). Thermal cycling conditions were, initially, 2 min at 50°C followed by 10 min at 95°C, and then, subsequently, 45 cycles of 15 s at 95°C and 1 min at 65°C.

Oligonucleotide primers and TaqMan probes were designed using Primer Express version 1.5 (Perkin-Elmer Applied Biosystems Inc.) and synthesized by Cybergene (Stockholm, Sweden) or ordered as a 'gene assay by demand' product (Perkin-Elmer Applied Biosystems Inc). The sequences or 'gene assay by demand' numbers can be provided on request. The probes were designed to cover exon-exon boundaries to avoid amplification of genomic DNA. As there are no predetermined low-abundance "house-keeping" genes for this experimental paradigm, the ΔΔCt method [51] was used to calculate relative changes in mRNA abundance. The threshold cycle (Ct) for 18S was subtracted from the Ct for the target gene to adjust for variations in mRNA/cDNA generation efficacy. This was carried out for both pre- and posttraining samples. The preexercise value reflects baseline gene expression levels and was subtracted from the postexercise value to calculate the increase or decrease in mRNA abundance.

A two-way mixed model ANOVA (GraphPad Prism 4.0) was used to establish whether a significant interaction between 'group' and 'extracellular gene responses' occurred (p < 0.0001) and to confirm appropriate baseline subject matching (p < 0.0001). Bonferoni post-hoc testing established which individual gene responses were significant for each subgroup, and this analysis was used for the basis of the discussion. For comparison between HRG and LRG training response data (e.g., peak VO_2_), a two-tailed unpaired t-test was utilized. Significance was accepted at the 5 % level. Data are mean ± SE.

## List of abbreviations used

α2-Macroglobulin (AM2)

Angiopoietin (ANG)

Angiopoietin 1 (ANG 1)

Angiopoietin 2 (ANG2)

Beats per minute (BPM)

Collagen type IIIα1 (COL3A1)

Collagen type XVα1 (COL15A1)

Extracellular matrix (ECM)

High-responder group (HRG)

Hypoxia-inducible factor 1α (HIF)

Interleukin 17D (IL17D)

Low responder group (LRG)

Myristoylated alanine-rich protein kinase C substrate (MARKS)

Neuropilin (NP-1)

Rate of oxygen consumption (VO_2_)

Rho-GTPase-activating protein 1 (ARHGAP1)

Threshold cycle (Ct)

Thrombospondin-4 (THBS4)

Transforming growth factor β (TGFB)

Transforming growth factor β2 (TGFB2)

Transforming growth factor β receptor II (TGFBR2)

Tyrosine kinase with immunoglobulin-like and EGF-like domains 1 (TIE1)

Tyrosine kinase with immunoglobulin-like and EGF-like domains 2 (TIE2)

Vascular endothelial growth factor (VEGF)

Vascular endothelial growth factor related receptor 1 (VEGFR1)

Vascular endothelial growth factor related receptor 2 (VEGFR2)

## Authors' contributions

EJ, JAT, PLG, JRa, JRi and CJS were responsible for the design and management of the endurance training study, subject recruitment and tissue sampling. JAT was responsible for generating the strategy for the gene expression analysis. JAT selected the biological theme and the majority of the genes to be profiled in this particular study, while TG selected some of the angiopoietin genes and ordered the majority of the primers. TG and HF were responsible for organizing the real time PCR analysis while JAT was responsible for the subsequent calculations and computations. JAT and CJS were responsible for drafting and revising the manuscript, respectively.

## Figures and Tables

**Table 1:** Baseline demographic and physiological parameters

	**LRG^1^**	**HRG^1^**	**p-value**
Height (cm)	180 ± 3	183 ± 3	p = 0.53
Age (year)	23 ± 1	24 ± 1	p = 0.54
Mass (kg)	77 ± 3	77 ± 6	p = 0.97
Mean blood pressure (mm Hg)	92 ± 1	88 ± 4	p = 0.25
Peak VO_2 _(L min^-1^)^2^	3.7 ± 0.1	3.5 ± 0.3	p = 0.48
Resting heart rate (BMP)	71 ± 5	70 ± 6	p = 0.94
Submaximal heart rate^3^	170 ± 5	171 ± 5	p = 0.85
Respiratory exchange ratio^4^	1.0 ± 0.0	1.0 ± 0.0	p = 0.45
15 min work (kJ)^5^	220 ± 9	204 ± 16	p = 0.37

^1^Values are mean (± SEM) taken prior to training.

^2^Peak VO_2 _is the increase in 'maximal' oxygen uptake measured during an incremental maximal exercise protocol.

^3^Submaximal heart rate was measured during 10 min constant load cycling at 75% peak VO_2_.

^4^The respiratory exchange ratio was obtained during 10 min of submaximal exercise at 75% of pretraining peak VO_2_.

^5^15 min work is the total work done in 15 min of self-paced cycling.
